# Supplementary material for: Comprehensive Analysis of the Impact of Weight Loss Thresholds on Mouse Models of Fatal Viral Infection
Source: Viruses. 2025 Sep 7;17(9):1225. doi: 10.3390/v17091225 (PMC12474137; doi:10.3390/v17091225)
Supplement: Supplementary file 1 [file viruses-17-01225-s001.zip › viruses-3824771-supplementary.pdf]

SUPPLEMENTAL INFORMATION
FOR
Comprehensive analysis of the impact of weight loss thresholds on mouse models of fatal viral infection

CONTENT:

1. Supplemental Figures and Legends (9 Supplemental Figures)

- **Figure S1.** Clinical scoring and mouse weight loss analysis in C57BL/6Ntac upon SARS-CoV-2 infection.
- **Figure S2.** Clinical scoring and mouse weight loss analysis in C57BL/6J upon SARS-CoV-2 infection.
- **Figure S3.** Low-dose SARS-CoV-2 infection of C57BL/6J mice.
- **Figure S4.** Clinical scoring and mouse weight loss analysis in BALB/c upon SARS-CoV-2 infection.
- **Figure S5.** Comparison of average weight in naïve male and female mice.
- **Figure S6.** Weight loss of survivors and non-survivors upon SARS-CoV-2 Delta and BA.1 infection.
- **Figure S7.** Clinical scoring of K18-hACE2 mice infected with SARS-CoV-2 Delta.
- **Figure S8.** Clinical scoring of K18-hACE2 mice infected with SARS-CoV-2 Omicron BA.1.
- **Figure S9.** Clinical scoring and mouse weight loss analysis upon Influenza A virus infection.

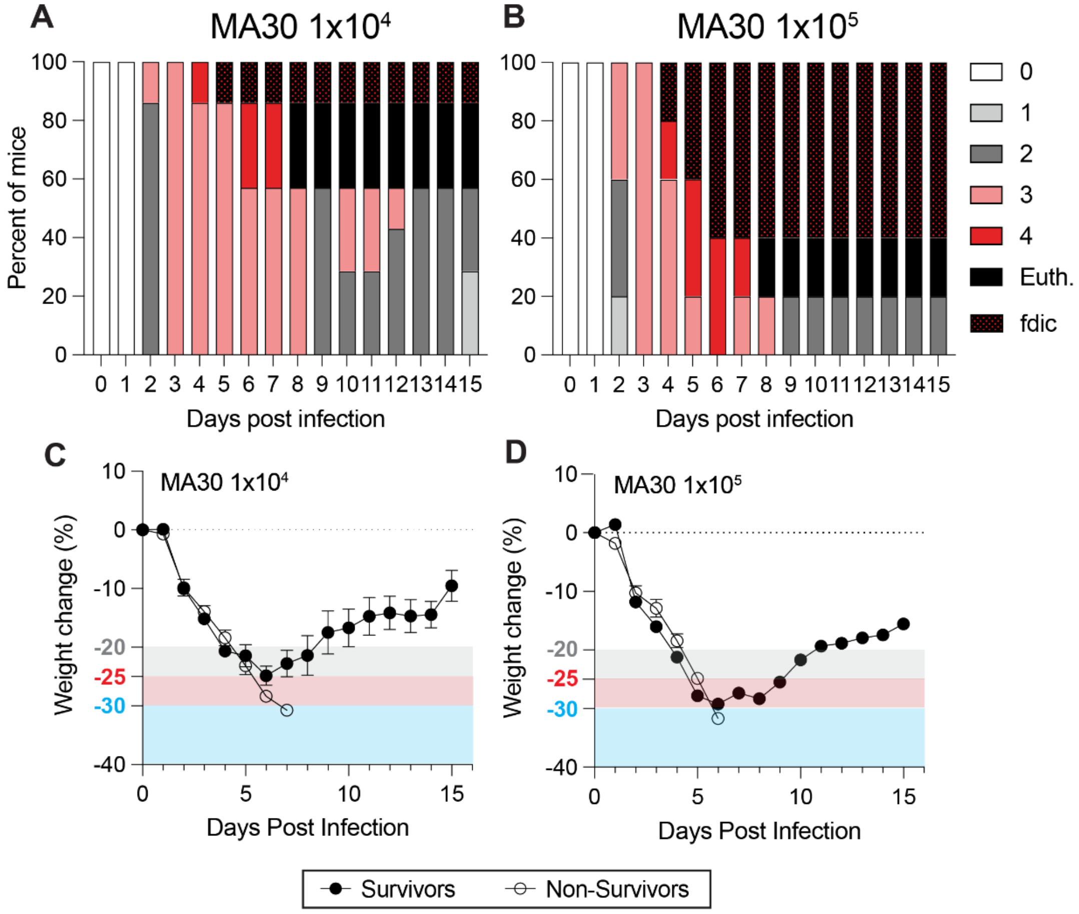

Figure S1. Clinical scoring and mouse weight loss analysis in C57BL/6Ntac upon SARS-CoV-2 infection. (**A–B**) Clinical scoring of C57BL/6Ntac mice challenged with (**A**) $1x10^4$ or (**B**) $1x10^5$ SARS-CoV-2 MA30 virus. Mice were assessed for weight change (10-30% weight loss), changes in appearance (hunched posture or ruffled fur), alterations in respiration (rapid respiration, shortened breath, etc.), reduced responsiveness, and neurological symptoms (head tilt, tremors, circling, etc.). Mice were scored on a binary system (0 or 1) for each category, daily scores were calculated, and cumulative scores were graphed as a percentage of the total mice. Mice that were found dead in cage (fdic) are indicated by red-dotted, black-filled portions and euthanized mice are shown as black-filled portions. (**C–D**) Weight loss of C57BL/6Ntac mice that survived (closed circle) or died (open circle) during infection with (**C**) $1x10^4$ and (**D**) $1x10^5$ PFU of SARS-CoV-2 MA30. A two-way ANOVA, mixed-effect model with Šídák's multiple comparison test was used to determine the significance of weight loss trends over the course of infection. If no p-value is shown between conditions, the difference is not statistically significant.

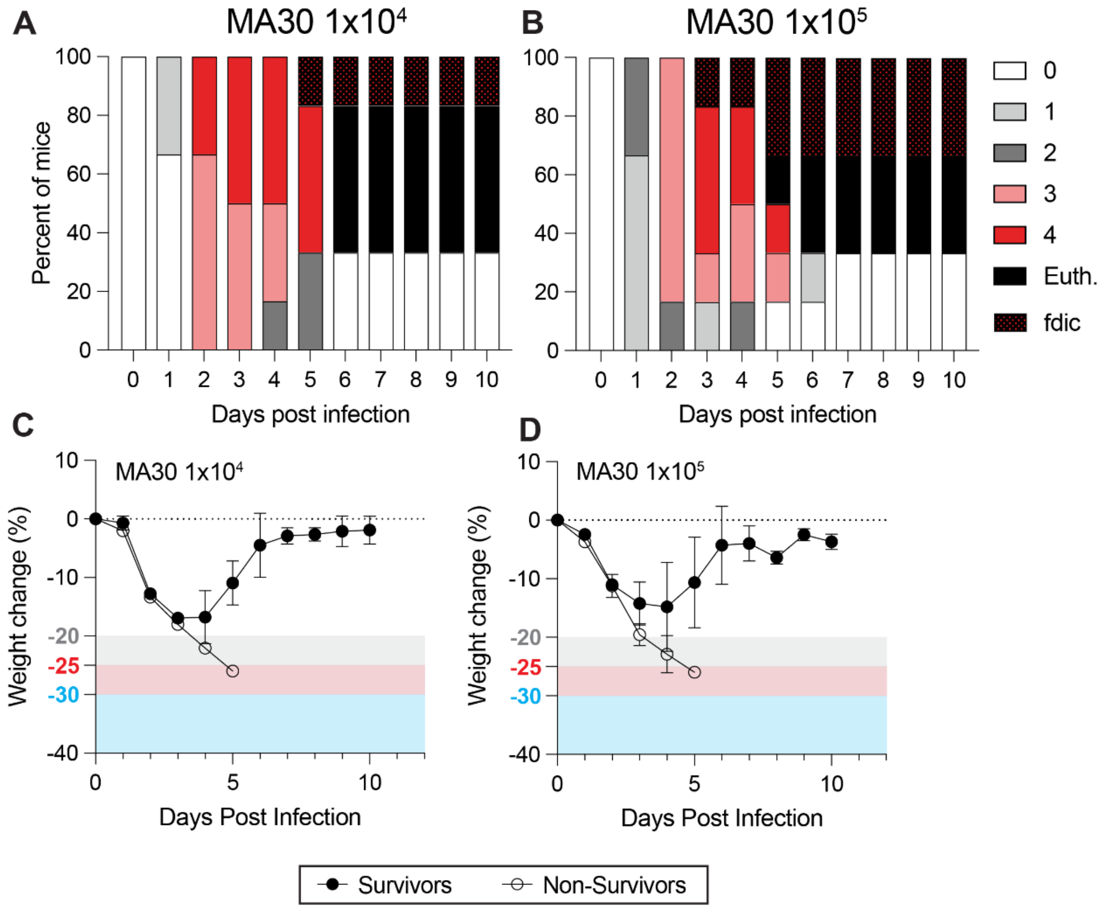

Figure S2. Clinical scoring and mouse weight loss analysis in C57BL/6J upon SARS-CoV-2 infection. (A–B) Clinical scoring of C57BL/6J mice challenged with (**A**) $1x10^4$ or (**B**) $1x10^5$ PFU of SARS-CoV-2 MA30 virus. Mice were assessed for weight change (10-30% weight loss), changes in appearance (hunched posture or ruffled fur), alterations in respiration (rapid respiration, shortened breath, etc.), reduced responsiveness, and neurological symptoms (head tilt, tremors, circling, etc.). Mice were scored on a binary system (0 or 1) for each category, daily scores were calculated, and cumulative scores were graphed as a percentage of the total mice. Mice that were fdic are indicated by red-dotted, black-filled portions and euthanized mice are shown as black-filled portions. (**C–D**) Weight loss of C57BL/6J mice that survived (closed circle) or died (open circle) during infection with (**C**) $1x10^4$ and (**D**) $1x10^5$ PFU of SARS-CoV-2 MA30. A two-way ANOVA, mixed-effect model with Šídák's multiple comparison test was used to determine the significance of weight loss trends over the course of infection. If no p-value is shown between conditions, the difference is not statistically significant.

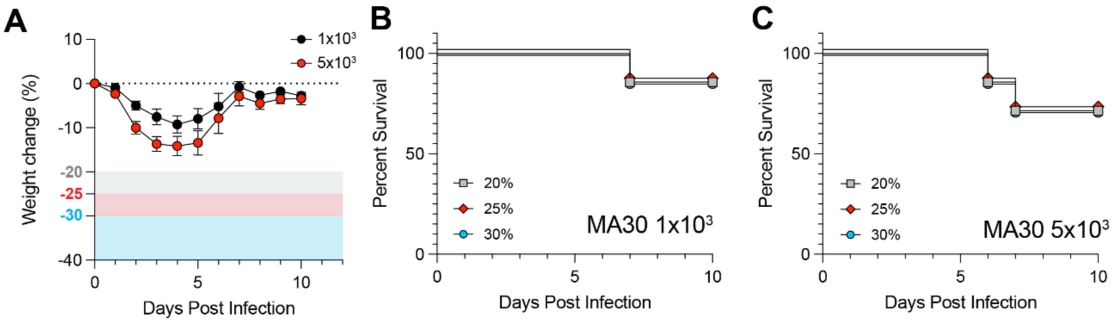

Figure S3. Low-dose SARS-CoV-2 infection of C57BL/6J mice. 13-22-week-old male and female C57BL/6J were infected with $1x10^3$ or $5x10^3$ PFU of SARS-CoV-2 MA30 virus. (**A**) Weight loss during infection with $1x10^3$ (black circles) or $5x10^3$ (red circles). Data are indicated as the mean ± SEM. (**B–C**) Survival plot of C57LB/6J infected with (**B**) $1x10^3$ or (**C**) $5x10^3$ PFU of SARS-CoV-2 MA30. A 20% (gray squares), 25% (red diamonds), or 30% (blue circles) WLT was applied. A Mantel–Cox log-rank test with a 95% confidence interval was applied to determine the significance of survival differences. A two-way ANOVA, mixed-effect model with Šídák's multiple comparison test was used to determine the significance of weight loss trends over the course of infection. If no p-value is shown between conditions, the difference is not statistically significant.

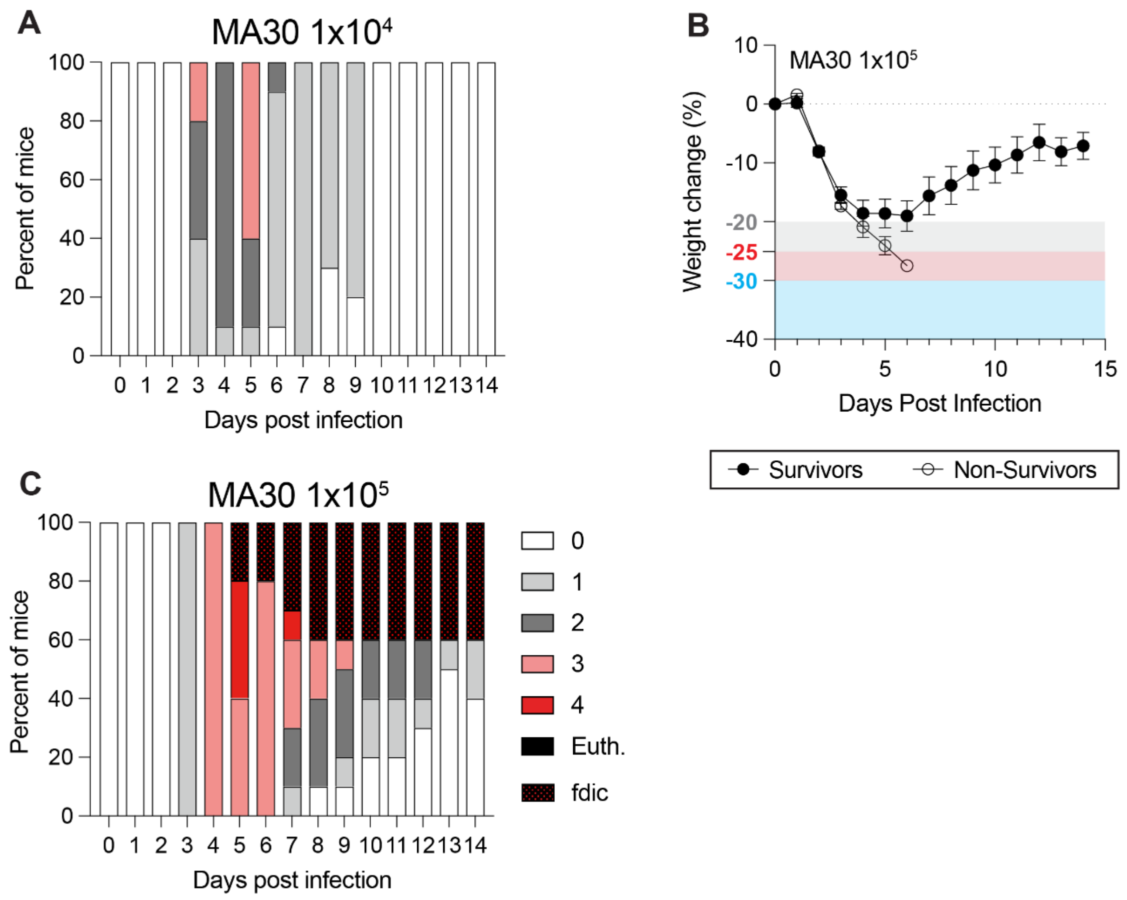

Figure S4. Clinical scoring and mouse weight loss analysis in BALB/c upon SARS-CoV-2 infection. (**A**) Clinical scoring of BALB/c challenged with $1x10^4$ PFU of SARS-CoV-2 MA30. Mice were assessed for weight change (10-30% weight loss), changes in appearance (hunched posture or ruffled fur), alterations in respiration (rapid respiration, shortened breath, etc.), reduced responsiveness, and neurological symptoms (head tilt, tremors, circling, etc.). Mice were scored on a binary system (0 or 1) for each category, daily scores were calculated, and cumulative scores were graphed as a percentage of the total mice. Mice that were fdic are indicated by red-dotted, black-filled portions and euthanized mice are shown as black-filled portions. (**B**) Weight loss in BALB/c mice that survived (closed circle) or died (open circle) during infection with $1x10^5$ PFU of SARS-CoV-2 MA30. (**C**) Clinical scoring of BALB/c challenged with $1x10^5$ PFU of SARS-CoV-2 MA30. Mice were assessed for weight change (10-30% weight loss), changes in appearance (hunched posture or ruffled fur), alterations in respiration (rapid respiration, shortened breath, etc.), reduced responsiveness, and neurological symptoms (head tilt, tremors, circling, etc.). Mice were scored on a binary system (0 or 1) for each category, daily scores were calculated, and cumulative scores were graphed as a percentage of the total mice. Mice that were fdic are indicated by red-dotted, black-filled portions and euthanized mice are shown as black-filled portions. A two-way ANOVA, mixed-effect model with Šídák's multiple comparison test was used to determine the significance of weight loss trends over the course of infection. If no p-value is shown between conditions, the difference is not statistically significant.

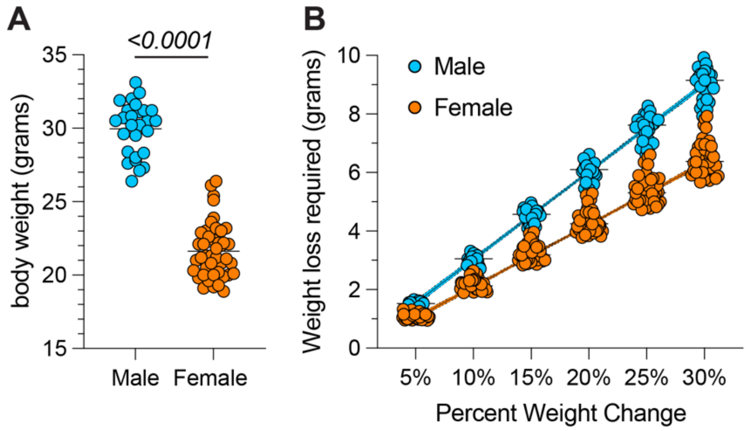

Figure S5. Comparison of average weight in naïve male and female mice. (**A**) Average initial weight of male (blue) and female (orange) mice, displayed as the mean ± SEM. Significance was determined by a two-tailed Welch's t-test. P-value is indicated on the graph. (**B**) Dot plot indicating the average weight loss required in grams (y-axis) for male (blue) and female (orange) mice to reach designated weight loss percentages (x-axis). Lines represent the linear regression with a 95% confidence interval.

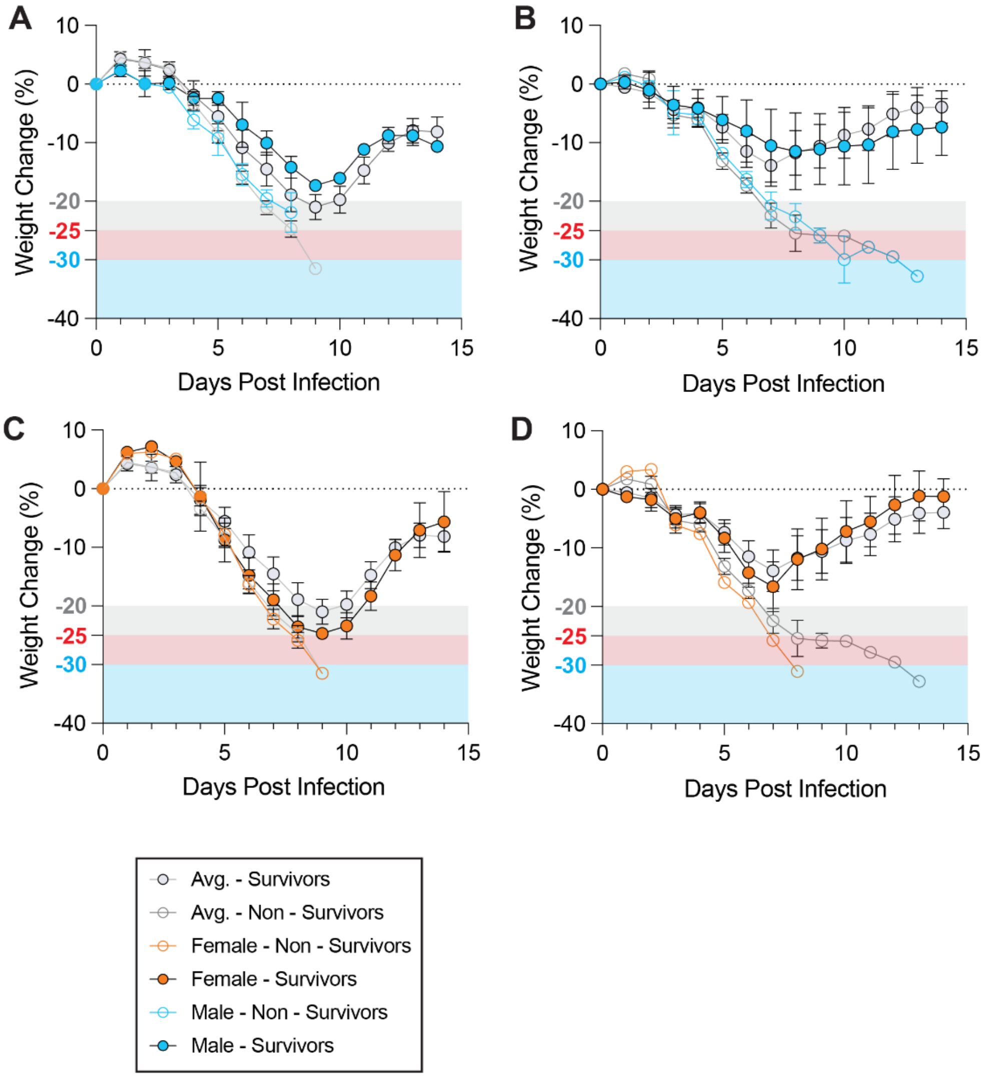

Figure S6. Weight loss of survivors and non-survivors upon SARS-CoV-2 Delta and BA.1 infection. (**A–D**) Weight loss in (**A–B**) male and (**C–D**) female K18-hACE2 mice during infection with (**A,C**) $1x10^4$ PFU of Delta or (**B,D**) $1x10^6$ PFU of Omicron BA.1. Mice that were either fdic or euthanized due to meeting euthanasia criteria are represented by open circles, and mice that survived the duration of the study are represented by closed circles. Average of male (blue) and female (orange) survivor (closed) and non-survivor (open) weight loss is indicated in gray. Data are indicated as the mean ± SEM. A two-way ANOVA, mixed-effect model with Šídák's multiple comparison test was used to determine the significance of weight loss trends over the course of infection. If no p-value is shown between conditions, the difference is not statistically significant.

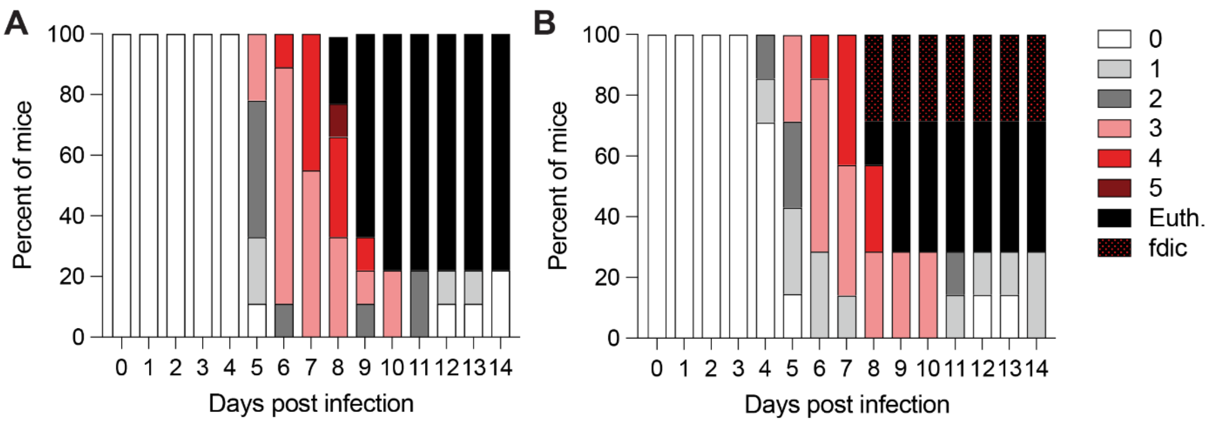

Figure S7. Clinical scoring of K18-hACE2 mice infected with SARS-CoV-2 Delta. (**A–B**) Clinical scoring of (**A**) female and (**B**) male mice challenged with 1x10^{4} PFU of Delta. Mice were assessed for weight change (10-30% weight loss), changes in appearance (hunched posture or ruffled fur), alterations in respiration (rapid respiration, shortened breath, etc.), reduced responsiveness, and neurological symptoms (head tilt, tremors, circling, etc.). Mice were scored on a binary system (0 or 1) for each category, daily scores were calculated, and cumulative scores were graphed as a percentage of the total mice. Mice that were fdic are indicated by red-dotted, black-filled portions and euthanized mice are shown as black-filled portions.

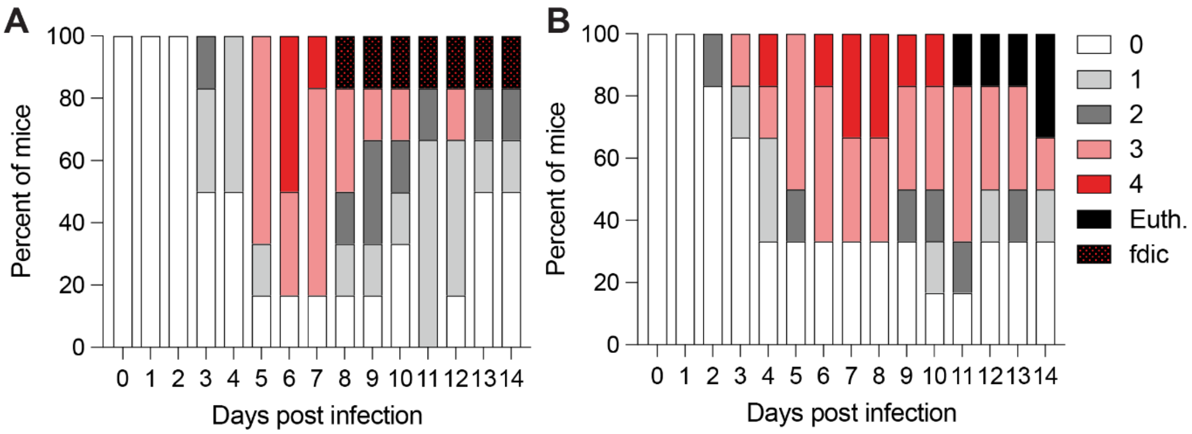

Figure S8. Clinical scoring of K18-hACE2 mice infected with SARS-CoV-2 Omicron BA.1. (**A–B**) Clinical scoring of (**A**) female and (**B**) male mice challenged with 1x10^{6} PFU of Omicron BA.1. Mice were assessed for weight change (10-30% weight loss), changes in appearance (hunched posture or ruffled fur), alterations in respiration (rapid respiration, shortened breath, etc.), reduced responsiveness, and neurological symptoms (head tilt, tremors, circling, etc.). Mice were scored on a binary system (0 or 1) for each category, daily scores were calculated, and cumulative scores were graphed as a percentage of the total mice. Mice that were fdic are indicated by red-dotted, black-filled portions and euthanized mice are shown as black-filled portions.

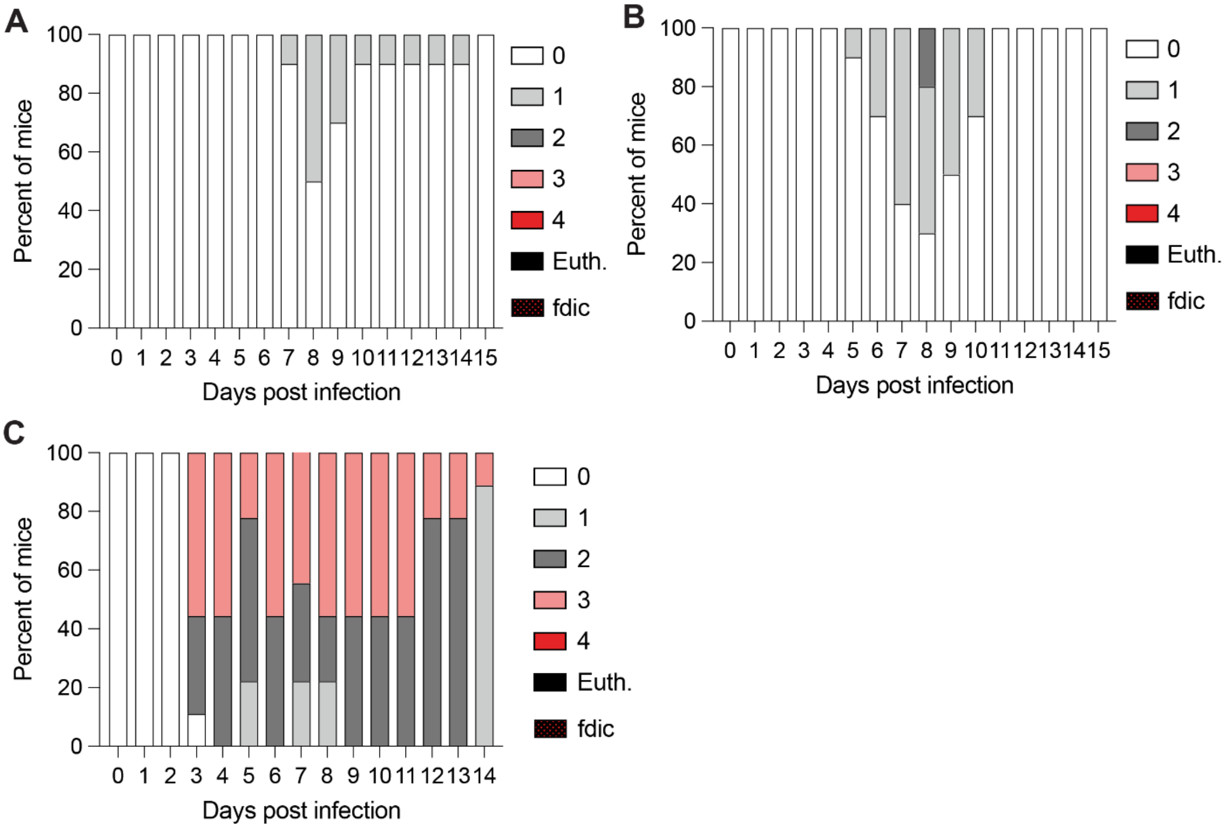

Figure S9. Clinical scoring and mouse weight loss analysis upon Influenza A virus infection. (**A–C**) Clinical scoring of C57BL/6J mice challenged with (**A**) 500 or (**B**) 1000 PFU of IAV PR8 or (**C**) BALB/c mice challenged with 1000 PFU of IAV PR8. Mice were assessed for weight change (10-30% weight loss), changes in appearance (hunched posture or ruffled fur), alterations in respiration (rapid respiration, shortened breath, etc.), reduced responsiveness, and neurological symptoms (head tilt, tremors, circling, etc.). Mice were scored on a binary system (0 or 1) for each category, daily scores were calculated, and cumulative scores were graphed as a percentage of the total mice. Mice that were fdic are indicated by red-dotted, black-filled portions and euthanized mice are shown as black-filled portions.
